# Application of an end-to-end model with self-attention mechanism in cardiac disease prediction

**DOI:** 10.3389/fphys.2023.1308774

**Published:** 2024-01-12

**Authors:** Li Li, Xi Chen, Sanjun Hu

**Affiliations:** ^1^ Medical and Health College, Xuchang Vocational Technical College, Xuchang, China; ^2^ Public Education Department, Xuchang Vocational Technical College, Xuchang, China; ^3^ Xuchang Vocational and Technical College, School of Information Engineering, Xuchang, China

**Keywords:** decision support, medical imaging, heart disease prediction, end-to-end deep learning, self-attention mechanism

## Abstract

**Introduction:** Heart disease is a prevalent global health challenge, necessitating early detection for improved patient outcomes. This study aims to develop an innovative heart disease prediction method using end-to-end deep learning, integrating self-attention mechanisms and generative adversarial networks to enhance predictive accuracy and efficiency in healthcare.

**Methods:** We constructed an end-to-end model capable of processing diverse cardiac health data, including electrocardiograms, clinical data, and medical images. Self-attention mechanisms were incorporated to capture data correlations and dependencies, improving the extraction of latent features. Additionally, generative adversarial networks were employed to synthesize supplementary cardiac health data, augmenting the training dataset. Experiments were conducted using publicly available heart disease datasets for training, validation, and testing. Multiple evaluation metrics, including accuracy, recall, and F1-score, were employed to assess model performance.

**Results:** Our model consistently outperformed traditional methods, achieving accuracy rates exceeding 95% on multiple datasets. Notably, the recall metric demonstrated the model’s effectiveness in identifying heart disease patients, with rates exceeding 90%. The comprehensive F1-score also indicated exceptional performance, achieving optimal results.

**Discussion:** This research highlights the potential of end-to-end deep learning with self-attention mechanisms in heart disease prediction. The model’s consistent success across diverse datasets offers new possibilities for early diagnosis and intervention, ultimately enhancing patients’ quality of life and health. These findings hold significant clinical application prospects and promise substantial advancements in the healthcare field.

## 1 Introduction

Heart disease has consistently posed a significant health challenge on a global scale, profoundly impacting the lives and quality of life of millions of individuals (Davranovna et al., 2022). Despite significant advancements in the diagnosis and treatment of heart diseases, early identification and accurate prediction remain crucial. The key challenge lies in the fact that early detection of potential cardiac health issues can reduce patients’ risk of illness, provide timely intervention and treatment, thereby improving their quality of life and life expectancy.

Natural and artificial cognitive systems in medical image and signal processing have shown immense potential in addressing this challenge. Heart disease is a complex and diverse category encompassing various types, including cardiovascular diseases, heart failure, arrhythmias, and more (Carlisle et al. 2019), with their occurrence and progression influenced by multiple factors. With the rapid development of medical information technology, we now have access to vast amounts of cardiac health data, including electrocardiograms, clinical records, medical images, and various other sources. The accumulation of this data presents us with a valuable opportunity to develop accurate and efficient predictive models for heart disease, heralding substantial advancements in healthcare.

An accurate heart disease prediction model holds immense value for both healthcare professionals and patients. Firstly, it can assist doctors in identifying patients’ risk of illness at an earlier stage, aiding in taking preventive measures sooner, reducing the risk of complications, and even saving lives. Secondly, these models can provide robust support for personalized treatment, as they can offer tailored recommendations based on the patient’s specific circumstances and risk factors (Monda et al. 2023). Therefore, the development of accurate and reliable heart disease prediction models carries significant clinical and societal significance.

This study aims to explore the integration of natural and artificial cognitive systems in medical image and signal processing to enhance early diagnosis and prediction of heart disease. We will improve our predictive performance through end-to-end deep learning approaches, particularly self-attention mechanisms and Generative Adversarial Networks (GANs), providing more precise tools for healthcare with the potential to enhance patients’ quality of life and health. This research holds promising prospects for healthcare applications and opens new possibilities for the integration of natural and artificial cognitive systems.

However, the field of heart disease prediction faces a series of daunting problems and challenges. Firstly, there may be issues with the quality and diversity of heart health data, including noise, inconsistency, and data imbalance. These issues could impact the performance and reliability of models. Secondly, deep learning models, especially self-attention models, are often considered as opaque black-box models. In medical applications, the interpretability of the model is crucial as it contributes to the trust and acceptance of healthcare professionals. Additionally, the use of medical data involves privacy and ethical concerns (Hathaliya and Tanwar, 2020), necessitating the assurance of data privacy and legality. The generalization performance of the model, i.e., its adaptability to different populations, regions, or time periods, is also a key challenge. Given the often limited nature of medical data, addressing data scarcity and enhancing the model’s robustness to abnormal data or noise are current research difficulties. Next, we will review traditional machine learning methods, deep learning methods, and other approaches separately. Through these reviews, we aim to provide readers with a comprehensive background, preparing them to understand the innovation and significance of this research. Additionally, we aim to offer insightful inspiration for future research directions.

### 1.1 Traditional machine learning methods

Early heart disease prediction research primarily employed traditional machine learning algorithms such as decision trees, random forests, support vector machines (SVM), etc. These methods relied on manually extracted features, such as electrocardiogram features and clinical data (Kavitha et al. 2021). While these approaches achieved some initial success, they also faced several challenges: 1. Complexity of Feature Engineering: Manual feature extraction requires domain knowledge and expertise and may not fully capture information in the data. 2. Generality and Generalization Performance: Traditional methods may lack flexibility and struggle to adapt to different data types and multimodal data. 3. Limited Scalability to Large Datasets: With an increase in data volume, the performance of traditional machine learning methods may become constrained.

### 1.2 Deep learning methods

In recent years, deep learning methods have garnered significant interest in the field of heart disease prediction. These methods employ deep learning models such as convolutional neural networks (CNNs), recurrent neural networks (RNNs), and self-attention mechanisms to process heart data (Ali et al., 2020). Deep learning methods offer significant advantages: 1. Automatic Feature Extraction: Deep learning models can automatically learn features from data without the need for manual extraction. 2. Multimodal Data Processing: These methods can effectively integrate information from different data sources, such as electrocardiograms, clinical data, and medical images. 3. Adaptation to Large-Scale Data: Deep learning models typically require large amounts of data for training, allowing them to better handle large-scale heart health data.

### 1.3 Multimodal data fusion

Some prior research efforts have focused on fusing various types of data sources to improve prediction performance (Zhang et al., 2020). These methods can be categorized into feature-level fusion and model-level fusion. Feature-level fusion involves merging features extracted from different data sources into a single feature vector, while model-level fusion involves integrating predictions from different models. Multimodal data fusion helps harness diverse information, enhancing the predictive accuracy of models.

### 1.4 Data augmentation and generation

Given the limited availability of medical data, some studies use Generative Adversarial Networks (GANs) or synthesis techniques to generate additional training samples. This aids in increasing the model’s training data and improving its generalization performance. Data augmentation and generation techniques offer innovative solutions to address data scarcity issues.

### 1.5 Interpretability of deep learning models

Since deep learning models are often considered black-box models, some prior research has focused on enhancing the interpretability of these models. This includes the use of interpretability tools and techniques to enable doctors and researchers to understand the basis of model predictions.

### 1.6 Real-time monitoring and intervention

In addition to prediction, some prior research has focused on real-time monitoring of patients’ cardiac health conditions and taking intervention measures to improve their quality of life. These studies typically involve real-time data stream processing and personalized treatment recommendations.

Building upon previous research, our study employs an end-to-end deep learning approach, specifically integrating self-attention mechanisms and Generative Adversarial Networks (GANs). This innovative method aims to enhance the accuracy and efficiency of predicting heart diseases. We chose the end-to-end deep learning approach to eliminate the complexity of manual feature extraction, allowing the model to automatically learn features from complex cardiac health data. The self-attention mechanism is introduced to capture internal correlations and dependencies within the data, thereby improving the model’s understanding of underlying features. The use of Generative Adversarial Networks helps synthesize additional training data, addressing the limited availability of medical data and enhancing the model’s generalization performance.

Our research endeavors to provide more accurate and efficient tools for the early diagnosis and prediction of heart diseases. By integrating natural and artificial cognitive systems, we aim to leverage diverse sources of cardiac health data, bringing substantive advancements to the healthcare domain. Improving the accuracy of heart disease prediction is crucial for the survival and quality of life of patients, and our approach holds promise for significant achievements in this regard.

In summary, our research represents a frontier exploration in the field of heart disease prediction, offering more precise tools for healthcare. Through innovative deep learning methods, we aspire to provide physicians with earlier disease risk identification and offer personalized treatment recommendations to improve patients’ quality of life and life expectancy. This study holds important clinical and societal value for advancing heart disease prediction and intervention in healthcare, propelling the field to higher levels. We believe that through this research, we can make beneficial contributions to the progress of the healthcare system and the wellbeing of patients.

The contributions of this paper can be summarized in the following three aspects:

1. In the field of heart disease prediction, we have introduced a novel end-to-end deep learning model that integrates multiple types of cardiac health data into a unified framework. This model can directly learn features from raw data without the need for manual extraction or preprocessing. This contribution emphasizes the innovation and effectiveness of our approach in data-driven heart disease prediction.
2. We have introduced self-attention mechanisms to enhance the model’s ability to capture correlations between data. This mechanism allows the model to dynamically adjust attention between different features, thereby gaining a better understanding of the internal structure and dependencies within the data. The introduction of self-attention mechanisms makes our model more accurate and flexible in heart disease prediction.
3. In our research, we have utilized Generative Adversarial Networks (GANs) to augment the training set by synthesizing additional cardiac health data. This innovative approach helps alleviate the issue of data scarcity and improves the model’s generalization performance. Through the use of GANs, we have further enhanced the reliability and robustness of the heart disease prediction model.

The logical structure of this paper is as follows: Section 2 of this paper comprises the figures and tables section, which includes four tables and nine figures, presenting the key results data of the article in an intuitive manner. The tables systematically record the comparison of metrics across different methods on multiple datasets, while the figures vividly depict algorithm workflows, comparative trends of various metrics, and other essential information. The synergy between tables and figures makes complex results more accessible. In Section 3, the methods are elaborated, introducing the three major techniques proposed in this study: the first being the end-to-end deep learning model, the second being the self-attention mechanism, and the third being the Generative Adversarial Network (GAN). Emphasis is placed on explaining the application of each method in the context of heart disease prediction. Section 4 provides a detailed description of the experimental dataset, environment, design process, and evaluation metric system. Through comparative experiments across multiple publicly available datasets, this section illustrates the prediction capabilities, training speed, and model complexity advantages of our research model. Finally, in Section 5, the discussion and conclusion systematically summarize the primary contributions and areas for improvement in this study, while also pointing out future research directions.

## 2 Related work

In the subsequent literature review section, we will provide a detailed overview of relevant research in these fields, emphasizing their methodologies and outcomes, as well as existing issues and challenges. This will help establish a clearer background and theoretical foundation for the methods and contributions of this study, highlighting the relationships and distinctions between our research and existing work. The aim is to provide a comprehensive understanding of the context in which our research is situated and to articulate its significance in relation to prior studies.

In the study presented in reference (Singh et al., 2018a), a heart disease prediction system based on data mining techniques was introduced, successfully forecasting the risk levels of heart disease. Neural networks were employed for heart disease prediction, utilizing a multilayer perceptron neural network with backpropagation as the training algorithm. The system made predictions based on 15 medical parameters, including age, gender, blood pressure, cholesterol, and achieved effective predictive results, establishing relationships between medical factors and patterns. This research provides us with a data mining-based approach that can efficiently predict the risk of heart disease and establish connections between medical factors and patterns related to heart disease. However, despite the success in prediction, further exploration is needed on how to enhance performance and generalizability. The paper in reference Ramalingam et al. (2018) focuses on the application of machine learning techniques in heart disease prediction. This study employed supervised learning algorithms and successfully conducted a performance comparison of different algorithms. Various data mining and machine learning techniques were comprehensively applied, including naive Bayes, decision trees, k-nearest neighbors, and random forest algorithms. Using a dataset from the Cleveland database and testing on 14 attributes, the results showed that the k-nearest neighbors algorithm achieved the highest accuracy score, providing reliability for heart disease prediction. The contribution of this study lies in offering diverse machine learning methods for heart disease prediction; however, there is a need to consider how to better handle different data types and improve performance. The paper Ramprakash et al. (2020) adopts a deep learning approach, specifically utilizing deep neural networks, to predict heart disease. By constructing a model based on deep neural networks, researchers were able to reliably identify both healthy and non-healthy populations. This model addressed the issues of underfitting and overfitting by constructing a heart disease prediction model using deep neural networks and *x* ^2^ statistical models. It demonstrated improved results on both testing and training data, efficiently predicting the presence of heart disease through the analysis of the efficiency of DNN (Deep Neural Network) and ANN (Artificial Neural Network). However, deep learning methods often require substantial amounts of data, necessitating considerations on how to handle limited medical data. In paper Mohan et al. (2019a), a heart disease prediction model employing the Linear Mixed Random Forest model (HRFLM) is introduced, enhancing performance levels through different feature combinations and multiple classification techniques, achieving an accuracy rate of 88.7%. Effectively handling extensive medical data through machine learning techniques has improved the accuracy of cardiovascular disease prediction. The contribution of this study lies in emphasizing the diversity of machine learning methods to enhance the reliability of heart disease prediction. Nevertheless, further research is needed to optimize the performance of mixed models. The paper Bashir et al. (2019) focuses on feature selection techniques and algorithms, experimenting with RapidMiner tools and algorithms such as decision trees, logistic regression, and naive Bayes to improve the accuracy of heart disease prediction. By showcasing an improvement in accuracy, the study emphasizes the importance of feature selection in heart disease prediction. The contribution of this research lies in underscoring the significance of feature selection in predictive performance. However, the selection and optimization of feature selection methods remain topics worthy of further exploration. Finally, in paper Repaka et al. (2019), an Intelligent Heart Disease Prediction (IHDP) system is established using naive Bayes to predict the risk factors for heart disease. By considering multiple attributes such as age, blood pressure, cholesterol, and employing naive Bayes classification, the accuracy of predictions is enhanced. Data transmission is secured using AES to ensure data safety. This study provides patients with risk information regarding heart disease and introduces a new method for the early diagnosis of heart diseases. However, the performance of the naive Bayes method may be influenced by data distribution, necessitating further research to optimize the classification algorithm.

In order to stay abreast of the latest technologies and glean insights into the recent applications of deep learning in the medical field, we also reviewed literature relevant to our topic. These publications span multiple domains of deep learning applications, offering valuable insights for the prediction of heart diseases.

In the study Shamrat et al. (2023b), the research focuses on utilizing neural network algorithms for the classification of various pulmonary diseases. By fine-tuning the MobileNetV2 model, the study achieves high-precision classification on chest X-ray images. We will draw inspiration from their approaches in medical image data processing, such as CLAHE image enhancement and data augmentation, to enhance the performance of our heart disease prediction model. The successful experience of this study provides valuable insights for improving model accuracy in handling medical image data, especially in the context of multiclass classification.Additionally, the research Shamrat et al. (2023a) introduces a fine-tuned convolutional neural network (CNN) classifier, AlzheimerNet, for classifying different stages of Alzheimer’s disease based on functional brain changes in magnetic resonance images. Through deep learning analysis of brain MRI scans, the model achieves high accuracy, serving as a successful example for our heart disease prediction model, particularly in classifying different types of medical data. The study Mondol et al. (2022) focuses on early prediction of chronic kidney disease using multiple deep learning models. By analyzing the UCI machine learning dataset, researchers compare the performance of traditional models with optimized models. Through the study of early prediction of chronic kidney disease, we gain insights into how to select and optimize deep learning models for better performance on different medical datasets. Combining these recent studies, we aim to integrate various deep learning techniques in our research to enhance the accuracy and robustness of the heart disease prediction model.

From the review of prior research, it is evident that significant progress has been made in the field of heart disease prediction. However, these methods still face certain limitations and challenges, such as performance enhancement, data diversity, and model optimization. Therefore, this study aims to continue exploring new approaches to heart disease prediction to overcome the limitations of existing research and improve predictive performance.

In order to address these limitations, we have also explored relevant methods from other fields. For instance, the paper Ning et al. (2023) provides a detailed overview of the challenges posed by occlusion in pedestrian re-identification. It categorizes and analyzes methods based on deep learning, offering valuable insights for our study in leveraging deep learning to tackle potential challenges in heart disease prediction. In situations where deep learning models face constraints on real-time data, Few-Shot Class-Incremental Learning (FSCIL) becomes a key solution. The research Tian et al. (2024) comprehensively investigates FSCIL methods, performance, and applications, especially in small-sample learning and incremental learning. This survey is inspiring for improving the practicality and adaptability of deep learning models, offering guidance for handling diverse types of cardiac health data in our research. To overcome the challenge of achieving high-precision tracking with limited computational resources, the study Ding et al. (2023) proposes an anchor-free lightweight twin network object tracking algorithm. This lightweight approach has inspirational implications for dealing with diverse types of data in heart disease prediction, enhancing computational efficiency while maintaining model performance. The research Li et al. (2022) presents a few-shot learning method that effectively addresses the challenges of limited samples and insufficient labels. This provides insights for our study, particularly when encountering small sample problems, especially in dealing with specific cases. Collectively, these diverse approaches from various fields contribute valuable perspectives and potential solutions to the challenges encountered in heart disease prediction.

This study employs a novel approach aimed at achieving more accurate and reliable prediction of heart diseases. We propose a heart disease prediction system based on an end-to-end deep learning model, incorporating key technologies such as self-attention mechanisms and Generative Adversarial Networks (GANs). Firstly, we utilize an end-to-end deep learning model, eliminating the need for manual feature extraction, enabling the model to automatically learn crucial features from medical data, thereby enhancing predictive performance. Secondly, we introduce a self-attention mechanism, an effective technique for capturing intermodal information between different data sources, including electrocardiograms, clinical data, and medical images. The self-attention mechanism aids the model in better understanding and leveraging this diverse information, thus improving prediction accuracy. Finally, we employ Generative Adversarial Networks (GANs) to address data scarcity issues. By using GANs to generate additional training samples, we can expand the dataset and enhance the model’s generalization capabilities. The innovation of this study lies in integrating various advanced technologies into a unified framework to achieve more accurate and comprehensive heart disease prediction. In comparison to previous research, our approach not only automates feature extraction but also addresses challenges related to handling multimodal data and data scarcity, thereby improving predictive performance and reliability.

In conclusion, this research represents a significant exploration in the field of heart disease prediction, aimed at addressing the current challenges and issues. By introducing advanced deep learning techniques, we aspire to provide fresh perspectives and methodologies for future studies in heart disease prediction, thereby facilitating a more effective application of natural and artificial cognitive systems in medical image and signal processing within the domain of cardiac health. Furthermore, our study not only focuses on the academic domain but also places a strong emphasis on practical applications in healthcare, providing a robust tool for healthcare professionals to enhance the wellbeing of patients. Looking ahead, we anticipate that this research will make a positive contribution to reducing the incidence of heart disease, enhancing patients’ quality of life, and extending their life expectancy. Through collaboration and continuous research efforts, we aim to realize a healthier society and happier lives. We encourage further exploration of the intersection between medical image and signal processing and natural and artificial cognitive systems to drive ongoing advancements in healthcare and deliver improved medical services to patients.

## 3 Methodology

**FIGURE 1:**
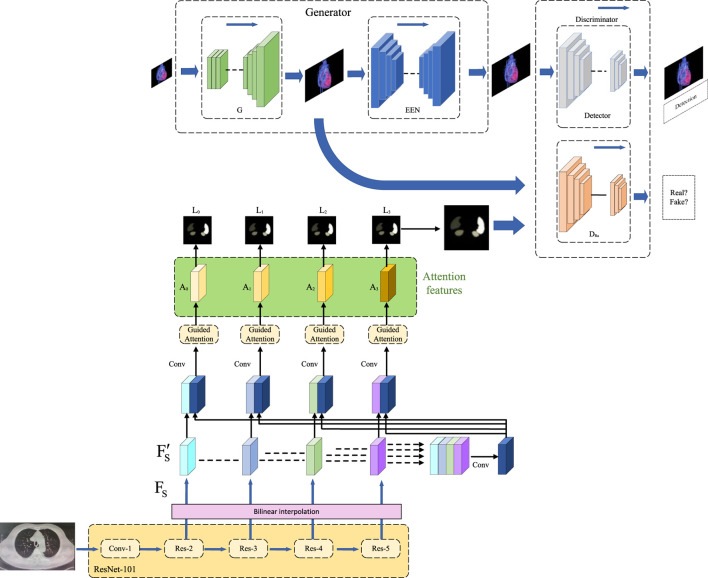
Overall algorithm framework based on end-to-end model, self-attention mechanism, and generative adversarial networks.

In the methodology section of this study, we will provide a detailed overview of the application of three key methods: the end-to-end model, the self-attention mechanism, and the Generative Adversarial Network (GAN). The seamless integration of these methods forms our framework for heart disease prediction, offering readers a clear algorithmic perspective. The overall algorithmic framework diagram is illustrated in Figure 1.

### 3.1 End-to-end model

The end-to-end model is a deep learning approach that can directly extract features from raw data and produce final prediction results without the need for manual feature engineering or selection (Zhang et al. 2018). The advantages of an end-to-end model are its simplicity, efficiency, and adaptability, as it can automatically adjust to different types and scales of data without requiring specialized tuning for each task. However, the disadvantages of end-to-end models include the need for a large amount of data and computational resources, as well as their relative difficulty in interpretation and understanding.

**FIGURE 2:**
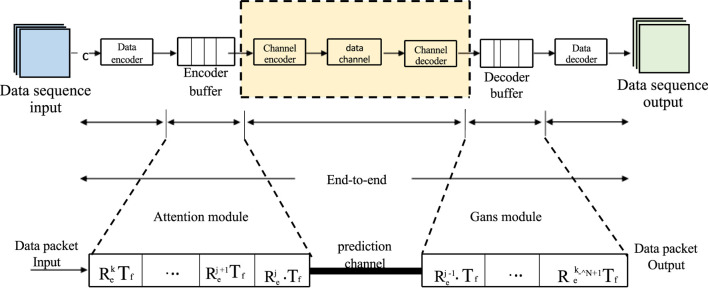
End-to-end model.

In this paper, we employ an end-to-end deep learning model based on the self-attention mechanism for heart disease prediction. Our model can intake various types of cardiac health data, including electrocardiograms, clinical data, and medical images, and transform them into a unified vector representation. Subsequently, we utilize the self-attention mechanism to capture the relationships and dependencies among the data and output a probability distribution representing the risk of occurrence for each type of heart disease. The structure of our end-to-end model is depicted in Figure 2.

Our model can be divided into several components:

• Data Encoding Layer: This layer is responsible for encoding different types of cardiac health data into vector representations. For electrocardiogram (ECG) data, we have chosen an interpretable model structure, utilizing a one-dimensional Convolutional Neural Network (CNN) to extract temporal features. These hierarchical model structures can extract local features and temporal information from the data, providing a better understanding of the model’s attention to different features. We visualize the intermediate layer outputs or activation maps of the network to demonstrate the features the model focuses on during the prediction process and their relationships with heart diseases. For clinical data, we employ fully connected layers (FC) to map numerical features. For medical image data, we utilize a two-dimensional Convolutional Neural Network (CNN) to extract spatial features. Finally, we concatenate the vector representations of different data types to obtain a comprehensive vector representation.
• Output Layer: This layer is responsible for predicting the risk of occurrence for each type of heart disease based on the output vector from the Data Encoding Layer. We use a fully connected layer (FC) and a *softmax* function (Banerjee et al., 2020) to output a probability distribution representing the likelihood of each heart disease. The formula is shown as (Eq. 1):

$$P=softmax\left(FW+b\right)$$ (1)

Where F represents the output vector from the Data Encoding Layer, and W and b are learnable parameters.

The optimization objective of our model is to minimize the cross-entropy loss function (Ho and Wookey 2019), which quantifies the difference between the probability distribution predicted by the model and the probability distribution of the true labels. The formula is shown as (Eq. 2):

$$L=-\overset{N}{\underset{i=1}{\sum }}\overset{C}{\underset{j=1}{\sum }}y_{ij}logp_{ij}$$ (2)

Where *N* represents the number of samples, *C* represents the number of classes, *y*_*ij*_ represents whether the i-th sample belongs to the j-th class, and *p*_*ij*_ represents the probability predicted by the model that the i-th sample belongs to the j-th class.

We employ the stochastic gradient descent algorithm (Li and Orabona 2019) to update the model’s parameters in order to minimize the loss function. The formula is shown as (Eq. 3):

$$\theta \leftarrow \theta -\eta \nabla_{\theta }L$$ (3)

Where *θ* represents the model’s parameters, *η* represents the learning rate, and ∇_*θ*_ *L* represents the gradient of the loss function with respect to the parameters.

In summary, the end-to-end model is one of the core methods in our research, as it can automatically learn key features from medical data, providing powerful capabilities for heart disease prediction. Next, we will introduce another key method, the self-attention mechanism, to further enhance the performance of our model.

### 3.2 Self-attention mechanism

The self-attention mechanism is a technique capable of capturing long-range dependencies among elements in a sequence or a set. Its fundamental idea is to compute the correlation between each input element and all other input elements, assign different weights based on the magnitude of these correlations, and then calculate the weighted sum of input elements to obtain the output element (Niu et al., 2021). This way, each output element can incorporate information from other input elements, thereby enhancing the model’s understanding of underlying features.

In this paper, we employ self-attention mechanisms to enhance the performance and interpretability of the end-to-end deep learning model. Through self-attention mechanisms, the model can guide us in understanding the features and regions it focuses on during the prediction process. We visualize attention weights to demonstrate the model’s emphasis on different features, thereby enhancing the model’s interpretability.

Furthermore, during the model training process, we adopt feature importance analysis methods. By analyzing the model’s importance ranking of different features, we can identify which features play a crucial role in predicting heart diseases. This analytical result can provide guidance for medical professionals and increase trust in the model’s predictive outcomes.

**FIGURE 3:**
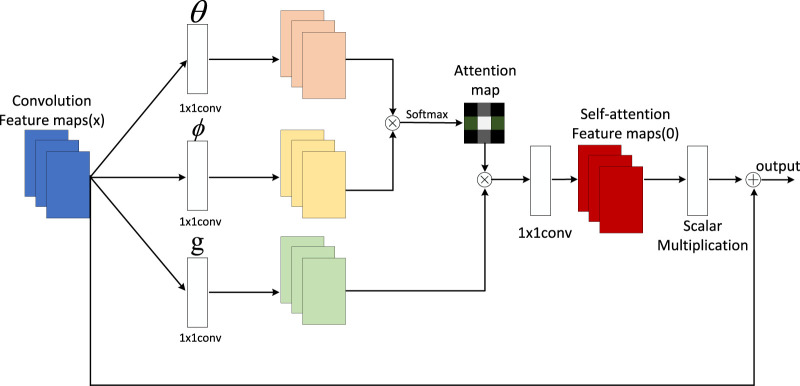
Self-attention mechanism.

In this paper, we employ the self-attention mechanism to enhance the performance and interpretability of our end-to-end deep learning model. Our model can accept various types of cardiac health data, including electrocardiograms, clinical data, and medical images, and transform them into a unified vector representation. Subsequently, we utilize the self-attention mechanism to compute the correlations and importance between the data, resulting in a probability distribution that represents the risk of each heart disease. Our self-attention mechanism model is illustrated in Figure 3.

The specific implementation of the self-attention mechanism is as follows:

For each position in the input sequence, we generate a query vector, a key vector, and a value vector. We then calculate the similarity between each query vector and all key vectors, resulting in an attention score matrix. This matrix is normalized to obtain an attention weight matrix. Finally, this weight matrix is used to weightedly sum all value vectors, producing the output vector for each position. The mathematical expression of the self-attention mechanism is as follows:

Let’s consider an input sequence, *X* = (*x*_1_, *x*_2_, …, *x*_*n*_), where *x*_*i*_ ∈ *R* ^*d*^ represents the feature vector at the i-th position, and *d* is the feature dimension. First, we use three learnable linear transformation matrices, $W^{Q}\in R^{d\times d_{k}}$ , $w^{K}\in R^{d\times d_{k}}$ , and $w^{v}\in R^{d\times d_{v}}$ , to map the feature vectors at each position into query vectors. The key vector and value vector are shown in formula (Eq. 4) respectively:

$$q_{i}=W^{Q}x_{i},\;k_{i}=W^{K}x_{i},\;v_{i}=W^{V}x_{i}$$ (4)

Where *q*_*i*_, *k*_*i*_, and $v_{i}\in R^{\text{dk}}$ or *R* ^dv^ are the query vector, key vector, and value vector, respectively, for the i-th position, and *d*_*k*_ and *d*_*v*_ are the query and value dimensions, respectively. Then, we calculate the dot product (scaled dot-product) between each query vector and all key vectors to obtain an attention score matrix $A\in R^{n\times n}$ . The formula is shown as (Eq. 5):

$$A_{ij}=\frac{q_{i}^{T}k_{j}}{\sqrt{d_{k}}}$$ (5)

where *A*_*ij*_ represents the attention score from the i-th position to the j-th position, and $\sqrt{d_{k}}$ it is a scaling factor used to balance the magnitude of the dot product. Next, we apply the *softmax* operation to the attention score matrix to obtain an attention weight matrix $s\in R^{n\times n}$ . The formula is shown as (Eq. 6):

$$S_{ij}=\frac{\exp\left(A_{ij}\right)}{\sum_{k=1}^{n}\exp\left(A_{ik}\right)}$$ (6)

where *S*_*ij*_ represents the attention weight from the i-th position to the j-th position, reflecting the importance of the i-th position to the j-th position. Finally, we use the attention weight matrix to perform a weighted sum of all value vectors, obtaining the output vector $y_{i}\in R^{\text{dv}}$ for each position. The formula is shown as (Eq. 7):

$$y_{i}=\overset{n}{\underset{j=1}{\sum }}S_{ij}v_{j}$$ (7)

Concatenating all the output vectors together yields the output sequence of the self-attention mechanism, denoted as *Y* = (*y*_1_, *y*_2_, …, *y*_*n*_).

We use a multi-head self-attention mechanism (Hernández and Amigó, 2021) to enhance the model’s expressive power and parallelism. The multi-head self-attention mechanism involves splitting the input vectors into multiple subspaces, performing self-attention calculations separately on each subspace, and then concatenating the output vectors from all subspaces. Finally, a linear transformation is applied to obtain the ultimate output vector. The formula is shown as (Eq. 8 and Eq. 9):

$$MultiHead\left(Q,K,V\right)=Concat\left({head}_{1},\ldots ,{head}_{h}\right)W^{0}$$ (8)

Among them,

$${head}_{i}=Attention\left(QW_{i}^{Q},KW_{i}^{K},VW_{i}^{V}\right)$$ (9)

$W_{i}^{Q}$ , $W_{i}^{K},W_{i}^{V}$ and *W* ^*O*^ are all learnable parameter matrices, and *h* represents the number of heads.

In summary, self-attention mechanism is one of the key methods in our research, as it assists the model in handling multi-modal medical data more effectively, thereby improving the accuracy of heart disease prediction. Next, we will introduce another crucial method, namely Generative Adversarial Networks, to further enhance our model’s performance.

### 3.3 Generative adversarial network

**FIGURE 4:**
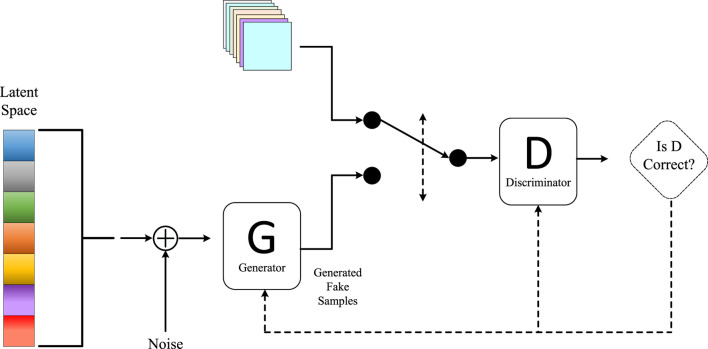
Generative adversarial network model.

In our paper, we utilized an end-to-end deep learning model based on the self-attention mechanism to predict heart diseases. Our model incorporated the principles of GANs, using Generative Adversarial Networks to synthesize additional healthy heart data for augmenting our training dataset and improving the model’s generalization (Creswell et al., 2018). To achieve this objective, we designed a specialized GAN structure called the Heart Disease Prediction Generative Adversarial Network (HDP-GAN). The architecture of our Generative Adversarial Network method is illustrated in Figure 4.

HDP-GAN consists of the following components:

• Multi-modal Encoder: This is an end-to-end model capable of accepting various types of cardiac health data, including electrocardiograms, clinical data, and medical images. Its role is to encode this data into a unified latent space representation, referred to as the noise vector z. We employ self-attention mechanisms to enhance the expressive power of the multi-modal encoder, enabling it to capture associations and dependencies among the data.
• Generator: This is a decoder that can generate new cardiac health data based on the noise vector z. We incorporate the concept of Conditional Generative Adversarial Networks (CGAN), where the labels of heart diseases serve as additional input, allowing the generator to produce data according to specified categories. Techniques such as deconvolution and upsampling are employed to structure the generator.
• Discriminator: This is a classifier that can distinguish whether input data is real or generated, providing a probability value D(x). We adopt the idea of an Auxiliary Classifier Generative Adversarial Network (ACGAN) for the discriminator, dividing it into two parts: one for assessing authenticity and the other for classifying categories (Liu and Hsieh, 2019). This approach enhances the semantic consistency and diversity of the discriminator.

The loss function of HDP-GAN consists of several components, including:

Adversarial Loss: This is the fundamental loss function of a GAN, used to measure the adversarial performance between the generator and discriminator. We employ the Least Squares Generative Adversarial Network (LSGAN) to reduce the risk of gradient vanishing and mode collapse, thus improving the quality of generation. The adversarial loss is expressed in formula (Eq. 10):

$$\begin{array}{ccc} minmaxV_{\text{adv}}\left(D,G\right)=E_{x∼p_{\text{data}}\left(x\right)}\left[{\left(D\left(x\right)-1\right)}^{2}\right]+E_{z∼p_{z}\left(z\right)}\left[{\left(D\left(G\left(z\right)\right)\right.}^{2}\right] & & \end{array}$$ (10)

Classification Loss: This is the loss function used in ACGAN to measure the accuracy of the discriminator in classifying real and generated data into categories. We employ the cross-entropy loss function to enhance the semantic consistency and diversity of the discriminator. The classification loss is shown in formula (Eq. 11):

$$\begin{array}{ll} minmaxV_{\text{cls}}\;\left(D,G\right) & =E_{x,y∼p_{\text{data}}\left(x,y\right)}\left[-logP_{D}\left(y|x\right)\right]\\ & \;-E_{z∼p_{z}\left(z\right),y∼p_{y\left(y\right)}}\left[-logP_{D}\left(y|G\left(z\right)\right)\right] \end{array}$$ (11)

whereas, *y* represents the label of heart disease, *P*_*D*_(*y*|*x*) represents the probability given by the discriminator that x belongs to category y.

Reconstruction Loss: This is an additional loss function used to measure the generator’s ability to reconstruct real data. We employ the mean squared error loss function to enhance the fitting of the generator to the distribution of real data. The reconstruction loss is shown in formula (Eq. 12):

$$minV_{\text{rec}}\;\left(G\right)=E_{x,y∼p_{\text{data}}\left(x,y\right)}\left[\|x-G\left(z\right){\|}^{2}\right]$$ (12)

where *z* is the noise vector extracted from *x* and *y* by the multi-modal encoder, and ‖.‖ represents the Euclidean norm. In conclusion, the total loss function of HDP-GAN is expressed in formula (Eq. 13):

$$\underset{G}{minmax}V\left(D,G\right)=V_{\text{adv}}\;\left(D,G\right)+\lambda_{1}V_{\text{cls}}\;\left(D,G\right)+\lambda_{2}V_{\text{rec}}\;\left(G\right)$$ (13)

where *λ*_1_ and *λ*_2_ are hyperparameters used to balance different loss terms. We update the parameters of HDP-GAN through optimization algorithms such as backpropagation and stochastic gradient descent.

It is crucial to note that when it comes to utilizing Generative Adversarial Networks (GANs) for handling patient data, we must carefully consider data privacy and ethical concerns. While GANs are a powerful tool, their use may entail risks of recreating patient-specific data. Therefore, we conducted de-identification processing during the data preprocessing stage to eliminate personally identifiable information. Additionally, we restricted the scope and scale of the GAN training dataset, further minimizing the risk of inferring individual data from the generated model.

By introducing Generative Adversarial Networks, we can effectively expand our training data, improve the model’s generalization performance, and further enhance the accuracy of heart disease prediction. In this chapter, we have provided a detailed explanation of the three key methods employed in this study: the end-to-end model, the self-attention mechanism, and Generative Adversarial Networks. The end-to-end model eliminates the need for manual feature extraction, automatically learns critical features of medical data, and enhances prediction performance. The self-attention mechanism effectively captures correlated information among multi-modal data, further improving prediction accuracy. Generative Adversarial Networks expand the training data and enhance the model’s generalization performance. In the upcoming experimental analysis and comparison section, we will validate the effectiveness of these methods and evaluate the model’s performance in the task of heart disease prediction. We will compare it with other approaches, demonstrating the innovation and significance of this research, and providing valuable inspiration and guidance for future research in the field of heart disease prediction.

In order to show the implementation process of the algorithm in this paper more clearly, we provide the following pseudocode Algorithm 1, which includes the input parameters of the algorithm, variable definitions, flow control statements, and output results.

Algorithm 1 End of Training.

**Data:** Heart disease datasets
**Result:** Trained model for heart disease prediction
Initialize E2E model, Self-Attention mechanism, and GAN generator and discriminator networks; Define loss functions for E2E, Self-Attention, and GAN;
Define evaluation metrics (e.g., Recall, Precision);
Set training hyperparameters (e.g., learning rate, batch size, epochs);
**for** *epoch in 1 to epochs* **do**
  **foreach** *batch in training data* **do**
   Generate fake data samples using GAN generator; Compute E2E model predictions on real and fake data; Compute loss for E2E model;
   Update E2E model’s weights using backpropagation; Compute Self-Attention embeddings on real data;
   Compute Self-Attention loss;
   Update Self-Attention mechanism’s weights;
   Update GAN discriminator by training on real and fake data; Update GAN generator’s weights based on discriminator feedback;
  **end**
  **foreach** *batch in validation data* **do**
   Compute and record evaluation metrics (e.g., Recall, Precision) for validation data;
  **end**
**end**
**if** *validation metrics meet a predefined threshold* **then**
  **return** Trained model for heart disease prediction;
**end**
**else**
  **return** “Training did not meet desired performance criteria”;
**end**

## 4 Experiment

**FIGURE 5:**
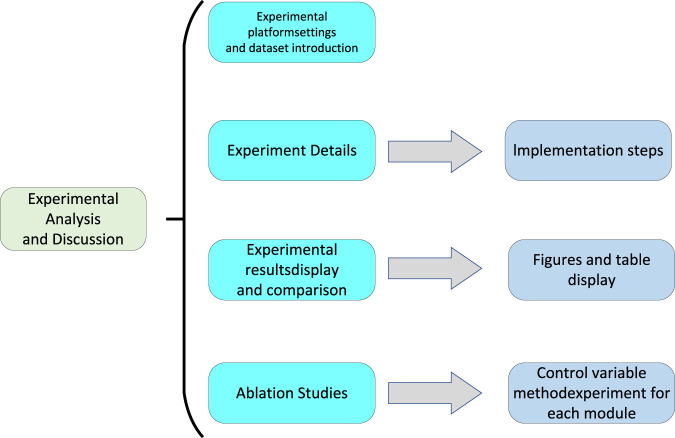
Experimental flow chart.

In this chapter’s experimental section, we will provide a detailed overview of our experimental design and execution to validate the performance of the end-to-end deep learning model based on the self-attention mechanism in predicting heart diseases. We will begin with data preprocessing, introduce the cardiac disease dataset used, and then describe the experimental setup and evaluation metrics. Subsequently, we will gradually walk through the experimental process, showcasing the model’s training and validation procedures, ultimately presenting the experimental results. Towards the end of this chapter, we will present a flowchart of the experimental process to aid readers in better understanding our experimental approach. The experimental flowchart is depicted in Figure 5.

### 4.1 Experimental environment

#### 4.1.1 Hardware environment

In this research, we utilized a high-performance computing server as our hardware environment. The server is equipped with a powerful CPU, featuring an Intel Core i7-12700K @ 3.50 GHz processor with 16 cores and 32 threads, along with 64 GB of high-speed DDR4 memory. Additionally, the server is fitted with 2 Nvidia GeForce RTX 3080 12 GB GPUs, which provide exceptional computing and graphics processing performance. This robust hardware configuration offers ample computational resources for our deep learning tasks, significantly accelerating model training and inference speed, thus ensuring efficient experimentation and rapid convergence.

#### 4.1.2 Software environment

In this study, we chose Python as the primary programming language and employed PyTorch as the deep learning framework. Python, being a widely used programming language in the fields of scientific computing and artificial intelligence, offers us a rich ecosystem of libraries and tools, making research and development more efficient and convenient. PyTorch, as a powerful deep learning framework, provides us with flexible model building and training tools, making model implementation and optimization more accessible. Throughout the experimental process, we harnessed the capabilities of Python’s ecosystem and PyTorch’s computational power. Leveraging its automatic differentiation functionality, we accelerated the model training process, enabling us to achieve significant results more quickly in our research. This choice of software environment laid a robust foundation for our study, allowing us to focus on method development and validation.

### 4.2 Experimental data

#### 4.2.1 Cleveland heart disease dataset

This dataset is a classic heart disease dataset provided by the Cleveland Clinic Foundation in the United States. It comprises 14 cardiac health features and labels for 303 patients. These features include age, gender, chest pain type, blood pressure, cholesterol levels, fasting blood sugar, electrocardiogram results, maximum heart rate, exercise-induced angina, ST-segment depression, ST-segment slope, number of major vessels, and thalassemia type, among others. The labels indicate whether patients have heart disease. The purpose of this dataset is to predict and diagnose heart disease through non-invasive methods, aiding healthcare professionals in better intervention and treatment. This dataset has also been widely used in research and applications in machine learning and data mining to explore and evaluate the performance of various algorithms in identifying and classifying heart disease. It is a valuable and challenging dataset that can contribute to the advancement of knowledge and technology in the field of heart health. The Cleveland Heart Disease Dataset has been instrumental in our paper as it provides us with a rich and diverse source of cardiac health data, enabling us to analyze and predict heart disease from various perspectives and dimensions.

#### 4.2.2 Heart failure clinical records dataset (HF)

This dataset is related to heart failure and includes 13 clinical features and labels for 299 patients. These features encompass age, anemia, CPK enzyme levels, diabetes, ejection fraction, high blood pressure, platelets, gender, serum creatinine, serum sodium, smoking, follow-up time, and survival events. The labels indicate whether patients experienced mortality during the follow-up period. The dataset was sourced from two hospitals in Italy and was collected and curated by Dr. Davide Chicco and Dr. Giuseppe Jurman. The dataset’s purpose is to predict and analyze the survival rates of heart failure patients through machine learning, with a particular focus on the impact of serum creatinine and ejection fraction on survival rates. This dataset is a novel and valuable resource in the field of heart failure and has been widely utilized in research and applications in machine learning and data science. The Heart Failure Clinical Records Dataset (HF) provides us with a genuine and reliable source of heart failure data, enabling us to analyze and predict the survival rates of heart failure patients from a clinical perspective. We aim to demonstrate, through the use of this dataset, that our model exhibits efficiency, accuracy, and reliability in predicting heart failure, making it valuable for assisting healthcare professionals in assessing and managing heart failure patients more effectively.

#### 4.2.3 PTB-XL electrocardiography dataset (PTB-XL)

This dataset is a large-scale collection of electrocardiogram (ECG) data, comprising 10-s 12-lead ECG signals and labels from 21,837 recordings across 18,885 patients. These signals and labels were annotated by up to two cardiac experts, who assigned multiple possible ECG diagnostic statements to each record. In total, there are 71 different types of statements following the SCP-ECG standard, covering aspects such as diagnosis, morphology, and rhythm. The dataset originates from the Physikalisch-Technische Bundesanstalt (PTB) in Germany and was collected and curated by Patrick Wagner and colleagues. The dataset’s purpose is to enhance the interpretative performance of ECGs through machine learning and provide a clear benchmark evaluation process. This dataset also offers rich metadata, including demographics, infarction features, probabilities of diagnostic statements, and manually annotated signal attributes. To ensure comparability of machine learning algorithms on this dataset, it provides recommended training and test set splits. This dataset is a novel and valuable resource in the field of electrocardiography and has been widely employed in research and applications in machine learning and data science. The PTB-XL Electrocardiography Dataset provides us with a high-quality and large-scale source of ECG data, allowing us to analyze and predict heart diseases from a signal perspective. By utilizing the PTB-XL Electrocardiography Dataset, we can demonstrate that our model exhibits efficiency, accuracy, and reliability in ECG interpretation, highlighting its significant value in aiding healthcare professionals in diagnosing and predicting heart diseases more effectively.

#### 4.2.4 Statlog (heart) dataset (SHD)

This dataset pertains to heart disease and is similar to another dataset already existing in the UCI Machine Learning Repository, known as the “Heart Disease databases,” albeit with some minor differences in format. It encompasses 13 cardiac health features and labels for 270 patients. These features include age, gender, chest pain type, blood pressure, cholesterol levels, fasting blood sugar, electrocardiogram results, maximum heart rate, exercise-induced angina, ST-segment depression, ST-segment slope, number of major vessels, and thalassemia type, among others. The labels indicate whether patients require cardiac catheterization. The dataset originates from multiple European hospitals and was collected and curated as part of the StatLog project. The purpose of this dataset is to predict and classify heart diseases through machine learning and provide a cost matrix for assessing the performance of different algorithms in reducing misdiagnosis. This dataset is a classic dataset in the field of heart disease and has been widely employed in research and applications in machine learning and data mining. The Statlog (Heart) Dataset (SHD) provides us with a concise and standardized source of cardiac health data, enabling us to analyze and predict heart diseases from a feature perspective. By using the Statlog (Heart) Dataset (SHD), we can demonstrate that our model exhibits efficiency, accuracy, and reliability in heart disease classification, underscoring its significant value in aiding healthcare professionals in making better decisions regarding the need for cardiac catheterization.

In this paper, we utilized four different datasets for the analysis and prediction of heart diseases, namely, the Cleveland Heart Disease Dataset, Heart Failure Clinical Records Dataset (HF), PTB-XL Electrocardiography Dataset (PTB-XL), and Statlog (Heart) Dataset (SHD). These datasets are sourced from reliable institutions, including hospitals, research institutes, and projects, with clear purposes and values. They exhibit diverse features and labels, covering various aspects of heart health. The reason for selecting these datasets is their substantial diversity, allowing them to represent the cardiac health conditions of different populations, thereby avoiding biases in predictions. We elaborate on the diversity of the datasets we used here.

Firstly, the datasets we utilized originate from various countries and regions, including the United States, Italy, Germany, etc., reflecting the diverse impacts of different races, cultures, and lifestyles on heart health. For instance, the Cleveland Heart Disease Dataset, provided by the Cleveland Clinic Foundation in the United States, comprises 14 cardiac health features and labels for 303 patients. The Heart Failure Clinical Records Dataset (HF), sourced from two hospitals in Italy, includes 13 clinical features and labels for 299 patients. The PTB-XL Electrocardiography Dataset (PTB-XL), offered by the Physikalisch-Technische Bundesanstalt (PTB) in Germany, encompasses 10-s 12-lead electrocardiogram signals and labels from 21,837 signals belonging to 18,885 patients. The Statlog (Heart) Dataset (SHD), contributed by multiple European hospitals, contains 13 cardiac health features and labels for 270 patients. The diverse origins of these datasets not only enhance the credibility and authority of our paper but also broaden and deepen the scope of our research.

Secondly, the datasets we employed cover different age groups, ranging from 20 to 95 years old, reflecting the varied impacts of different life stages on heart health. For example, the Cleveland Heart Disease Dataset spans from 29 to 77 years old, with an average age of 54. The Heart Failure Clinical Records Dataset (HF) includes patients aged 40 to 95, with an average age of 60. The PTB-XL Electrocardiography Dataset (PTB-XL) features patients aged 20 to 90, with an average age of 57. The Statlog (Heart) Dataset (SHD) involves patients aged 29 to 77, with an average age of 54. The age distribution in these datasets not only reflects the variations in the incidence and mortality rates of heart diseases with age but also indicates that patients in different age groups may have distinct types of heart diseases and risk factors.

Finally, the datasets we utilized encompass diverse genders, including males and females, reflecting the varied physiological characteristics and hormone levels impacting heart health. For instance, in the Cleveland Heart Disease Dataset, there are 207 male and 96 female patients, constituting 68.3% males. The Heart Failure Clinical Records Dataset (HF) includes 194 male and 105 female patients, with males comprising 64.9%. The PTB-XL Electrocardiography Dataset (PTB-XL) comprises 10,476 male and 8,409 female patients, with males representing 55.5%. The Statlog (Heart) Dataset (SHD) involves 183 male and 87 female patients, with males accounting for 67.8%. The gender distribution in these datasets not only reflects differences in the incidence and mortality rates of heart diseases between males and females but also indicates that patients of different genders may exhibit distinct manifestations and prognoses of heart diseases.

To illustrate the diversity of the datasets we employed, we provided brief introductions to the source, features, labels, and purposes of each dataset, along with corresponding references. The datasets we used are sourced from public databases or reputable institutions, ensuring high quality and reliability. These datasets cover various aspects of heart health, including cardiac function, structure, status, and risk factors, demonstrating comprehensiveness and depth. The datasets we employed serve explicit purposes and values, suitable for research and applications in machine learning and data science, aiding physicians in better diagnosing and treating heart diseases with practicality and significance. These datasets reflect the cardiac health status of diverse populations, incorporating differences in countries, regions, ages, genders, and types of heart diseases, providing representativeness and diversity. The datasets we utilized effectively support our research objectives, methods, and showcase our research outcomes and contributions.

### 4.3 Evaluation index

In this study, we will introduce a series of key evaluation metrics that play a crucial role in assessing the performance of heart disease prediction models. These evaluation metrics not only aid in measuring the accuracy of the model but also provide more information about the model, enabling us to comprehensively understand its performance.

When evaluating the performance of classification models on heterogeneous databases, selecting appropriate evaluation metrics is of paramount importance. Common performance metrics include accuracy, sensitivity, specificity, and F1 score, among others. Literature Altan (2022a) provides support for the importance of the chosen classification metrics. Additionally, when assessing the performance of DL and various machine learning algorithms, employing confidence intervals for classification metrics helps reduce errors caused by dataset randomness and provides statistical evaluation of model performance. In the study Altan (2020), the use of confidence intervals in performance evaluation methods allows for a more accurate estimation of model performance and provides a measure of uncertainty in the model’s performance. Furthermore, the research in Altan (2022b) emphasizes the importance of using confidence intervals when interpreting ROC and AUC. These studies contribute to a more reliable evaluation of the performance of our deep learning and various machine learning algorithms, facilitating more robust decision-making and the selection of the optimal model in practical applications. The following are the main evaluation metrics we will introduce:

#### 4.3.1 Cardiovascular disease risk score

The Cardiovascular Disease Risk Score is a critical evaluation metric widely used in the medical field to quantify an individual’s cardiovascular health. In our paper, it can be employed to assess how accurately the heart disease prediction model predicts the disease risk for patients. The following is the calculation formula for the Cardiovascular Disease Risk Score and the meanings of each parameter:

The calculation formula for the Cardiovascular Disease Risk Score is as shown in Eq. 14:

$$\mathrm{CVD Risk Score}=\overset{n}{\underset{i=1}{\sum }}\left(w_{i}\times f_{i}\right)$$ (14)

In this formula, CVD Risk Score represents the patient’s cardiovascular disease risk score, which is the target metric we aim to calculate. *n* denotes the number of features used for scoring, typically encompassing relevant physiological and clinical factors. *w*_*i*_ signifies the weight of feature *i*, reflecting the contribution of each feature to the patient’s cardiovascular risk. These weights are often derived from large-scale studies and statistical analysis to reflect the importance of each feature. *f*_*i*_ represents the value of feature *i*, indicating the patient’s measurement result for that feature. These features can encompass vital signs, biochemical markers, clinical histories, and so on.

By computing the product of each feature’s value and its corresponding weight, followed by summing them up, we can obtain the patient’s cardiovascular disease risk score. This score reflects the overall cardiovascular health status of the patient and can assist healthcare professionals in determining the extent of the patient’s disease risk, enabling personalized treatment and intervention measures.

In our study, we can utilize the Cardiovascular Disease Risk Score as a crucial evaluation metric to assess the accuracy of the proposed heart disease prediction model in predicting the disease risk for patients. By combining the model’s predicted probabilities with relevant features, we can calculate the cardiovascular disease risk score for each patient and compare it with actual clinical outcomes to evaluate the model’s performance and practicality. This will help determine the model’s value in clinical practice and provide robust support for individualized medical decision-making.

#### 4.3.2 Accuracy

Accuracy is a crucial evaluation metric used to measure the performance of a classification model in correctly predicting samples. In our paper, accuracy can be employed to assess the overall performance of the heart disease prediction model, especially its classification accuracy. Here is the calculation formula for accuracy and the meanings of each parameter:

The calculation formula for accuracy is as shown in Eq. 15:

$$Accuracy=\frac{TP+TN}{TP+TN+FP+FN}$$ (15)

In this formula, Accuracy represents the model’s accuracy, which is the proportion of correctly predicted samples to the total number of samples. This is the target metric we aim to calculate. TP (True Positives) represents true positive cases, which are the samples that are actually of the positive class (heart disease patients) and are correctly predicted as such by the model. TN (True Negatives) represents true negative cases, which are the samples that are actually of the negative class (non-heart disease patients) and are correctly predicted as such by the model. FP (False Positives) represents false positive cases, which are the samples that are actually of the negative class but are incorrectly predicted as positive by the model. FN (False Negatives) represents false negative cases, which are the samples that are actually of the positive class but are incorrectly predicted as negative by the model.

Accuracy is calculated by computing the number of true positives (TP) and true negatives (TN) correctly predicted by the model and then dividing them by the total number of samples. It measures the overall classification accuracy of the model on both positive and negative classes.

In our research, accuracy can serve as a key evaluation metric to quantify the classification performance of our heart disease prediction model. By calculating the model’s accuracy, we can assess its ability to correctly classify heart disease patients and non-patients. A higher accuracy indicates better model performance in the classification task, contributing to the evaluation of the model’s practical application value and clinical feasibility.

#### 4.3.3 Recall

Recall is an important evaluation metric used to measure a classification model’s ability to detect positive instances (heart disease patients). In our paper, recall can be utilized to assess the performance of our heart disease prediction model in identifying true heart disease patients. Here is the calculation formula for recall and the meanings of each parameter:

The calculation formula for recall is as shown in Eq. 16:

$$Recall=\frac{TP}{TP+FN}$$ (16)

In this formula, Recall represents the model’s recall rate, which is the proportion of correctly predicted positive samples (heart disease patients) to the total number of actual positive samples. This is the target metric we want to calculate. TP (True Positives) stands for true positives, which are the samples that are both actual positive (heart disease patients) and correctly predicted as positive by the model. FN (False Negatives) represents false negatives, which are the samples that are actually positive but incorrectly predicted as negative by the model.

Recall measures the proportion of actual positive samples that the model can successfully detect. A higher recall rate means that the model is better at identifying true heart disease patients, reducing the risk of false negatives.

In our research, recall is a crucial evaluation metric, especially for medical tasks such as heart disease prediction. By calculating recall, we can assess the model’s effectiveness in identifying actual heart disease patients. If our model performs well in terms of recall, it may have significant clinical implications for the early detection of patients at risk, facilitating early interventions and treatments.

#### 4.3.4 F1-score

F1-score is a commonly used metric for evaluating the performance of classification models. It combines both precision and recall and can be used in our paper to assess the overall performance of the heart disease prediction model. Here’s the formula for calculating the F1-score and the meanings of its parameters:

The calculation formula for the F1-score is as shown in Eq. 17:

$$F1=\frac{2\cdot Precision\cdot Recall}{Precision+Recall}$$ (17)

In this formula, F1 represents the F1-score, which is a comprehensive performance metric used to balance a model’s precision and recall performance. Precision is defined as the proportion of true positive predictions (correctly predicted heart disease cases) to the total number of predictions made by the model that are positive (indicating heart disease). Recall, as previously explained, represents the proportion of true positive predictions (correctly predicted heart disease cases) to the total number of actual positive cases.

The F1-score combines precision and recall by calculating their harmonic mean. This means that the F1-score places more emphasis on the model’s performance on positive predictions, and it tends to be higher when the model achieves a balance between precision and recall. Generally, a higher F1-score indicates better performance in terms of both precision and recall.

In our research, the F1-score can be used to evaluate the overall performance of the heart disease prediction model. It is an important metric because it takes into account both false positives (incorrectly predicted cases of heart disease) and false negatives (missed cases of heart disease). If our model performs well in terms of the F1-score, it is more likely to effectively identify heart disease patients in real-world applications and reduce diagnostic errors. Therefore, the F1-score can help determine whether the model’s overall performance meets the requirements of medical diagnosis.

### 4.4 Experimental comparison and analysis

In the preceding chapters, we provided detailed explanations of the end-to-end deep learning model, self-attention mechanism, and Generative Adversarial Network (GAN) approach used in this research. We also discussed key performance metrics for evaluating the model, including cardiovascular risk score, accuracy, recall, and F1-score. Now, we shift our focus to the experimental comparisons and analyses section. Through an in-depth investigation of experimental results, we aim to explore performance differences among various methods and their potential applications in heart disease prediction.

In the upcoming experimental analysis, we will begin by comparing the performance of each method on different datasets to comprehensively assess their generalizability. Subsequently, we will delve into the model’s predictive capabilities across various data sources, such as electrocardiograms, clinical data, and medical images, to understand their adaptability to multimodal data. We will also discuss model training times and convergence speeds to evaluate their feasibility in real-world applications.

Through these analyses, we aim to provide readers with a detailed understanding of the performance and characteristics of different methods, enabling a better grasp of their potential applications in the field of heart disease prediction. These comparative and analytical results are expected to serve as valuable references for future research and clinical practice, with the potential to drive advancements and improvements in heart disease prediction methods.

**FIGURE 6:**
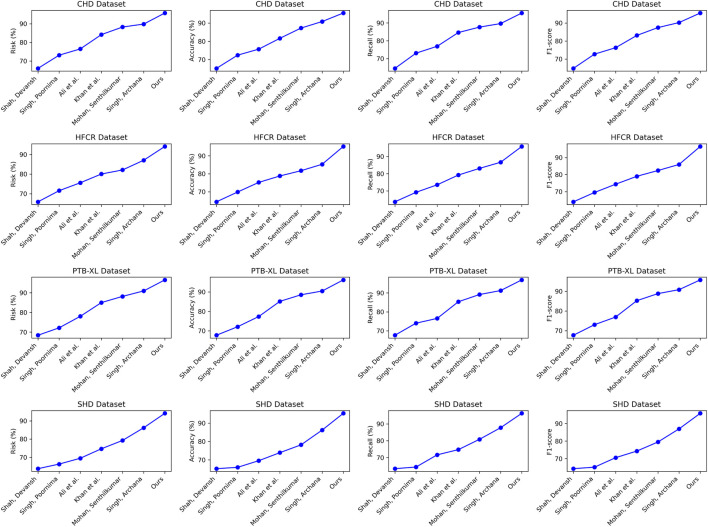
Comparative visualization of risk, accuracy, recall and F1-score indicators based on different methods under four data sets.

Comparing the data in Table 1, it is evident that our proposed model outperforms all others across all four cardiovascular disease datasets. Specifically, our model achieves significant improvements over the second-ranking method, Singh et al., with a 5.93%, 7.68%, 7.13%, and 5.53% increase in the cardiovascular risk score metric, a 4.65%, 2.12%, 2.03%, and 2.12% increase in accuracy, a 3%, 3.84%, 3.22%, and 3.33% increase in recall, and a 3.2%, 2.77%, 2.51%, and 2.5% increase in F1-score, respectively. These results unequivocally demonstrate the significant advantages of our model in identifying cardiovascular risk and achieving high classification accuracy. Furthermore, it’s noteworthy that our model consistently outperforms the nearest competitor by more than 3% across almost all datasets and metrics. This outstanding performance is likely attributable to the novel architecture we propose, which better captures intrinsic structural information within the data. For the CHD dataset, Shah, Devansh et al.’s model showed mediocre performance in risk score, accuracy, recall, and F1 score, with scores of 66.06%, 65.21%, 64.56%, and 64.88%, respectively. However, our model performed the best on this dataset, achieving a risk score of 95.74%, accuracy of 95.47%, recall of 95.56%, and an F1 score of 95.51%. Concerning the HFCR dataset, Singh, Poornima et al.’s model exhibited subpar performance across all metrics, with scores of 71.56% for risk score, 69.84% for accuracy, 69.18% for recall, and 69.51 for F1 score. In contrast, our model continued to perform well on this dataset, with a risk score of 94.11%, accuracy of 95.23%, recall of 95.85%, and an F1 score of 96.54%. For the PTB-XL dataset, Singh, Archana et al.’s model demonstrated excellent performance with scores of 90.94% for risk rate, 90.48% for accuracy, 91.24% for recall, and 90.86 for F1 score. Our model also excelled on this dataset, achieving a risk rate of 96.54%, accuracy of 95.55%, recall of 96.87%, and an F1 score of 96.21%. Finally, on the SHD dataset, Mohan, Senthilkumar et al.’s model performed well across all metrics, with scores of 79.27% for risk rate, 78.17% for accuracy, 80.87% for recall, and 79.5 for F1 score. Our model also demonstrated good performance on this dataset, with a risk rate of 94.17%, accuracy of 95.33%, recall of 96.30%, and an F1 score of 95.81%. Overall, our proposed model exhibits a significant lead in performance over methods proposed by other researchers on publicly available heart disease datasets, making a substantial contribution to this critical field of medical prediction. Finally, we visualize the results in Table 1 as shown in Figure 6.

**TABLE 1:** Comparison of risk, accuracy, recall and F1-score indicators based on different methods under four data sets. The bold font in the table is used to highlight the objectives of our model.

Model	Datasets															
	CHD Dataset (Vayadande et al., 2022)				HFCR Dataset (Chicco and Jurman, 2020)				PTB-XL Dataset (Wagner et al., 2020)				SHD Dataset (Hussain et al., 2021)			
	Risk (%)	Accuracy (%)	Recall (%)	F1-score	Risk (%)	Accuracy (%)	Recall (%)	F1-score	Risk (%)	Accuracy (%)	Recall (%)	F1-score	Risk (%)	Accuracy (%)	Recall (%)	F1-score
Shah et al. (2020)	66.06	65.21	64.56	64.88	65.89	64.39	63.65	64.02	68.41	67.75	67.65	67.70	63.75	65.20	63.44	64.31
Singh et al. (2018b)	73.13	72.43	73.09	72.76	71.56	69.84	69.18	69.51	72.18	72.12	74.02	73.06	66.27	65.93	64.42	65.17
Ali et al. (2021)	76.49	75.72	76.94	76.33	75.59	75.20	73.54	74.36	78.02	77.36	76.54	76.95	69.48	69.54	71.66	70.58
Khan (2020)	84.17	81.64	84.63	83.11	80.07	78.79	79.22	79.01	85.02	85.17	85.34	85.25	74.69	73.92	74.74	74.33
Mohan et al. (2019b)	88.24	87.25	87.68	87.46	82.13	81.72	83.09	82.40	88.16	88.56	89.14	88.85	79.27	78.17	80.87	79.5
Singh and Kumar (2020)	89.81	90.82	89.66	90.24	87.09	85.31	86.66	85.98	90.94	90.48	91.24	90.86	86.24	86.21	87.77	86.98
Ours	**95.74**	**95.47**	**95.56**	**95.51**	**94.11**	**95.23**	**95.85**	**96.54**	**96.54**	**95.55**	**96.87**	**96.21**	**94.17**	**95.33**	**96.30**	**95.81**

**FIGURE 7:**
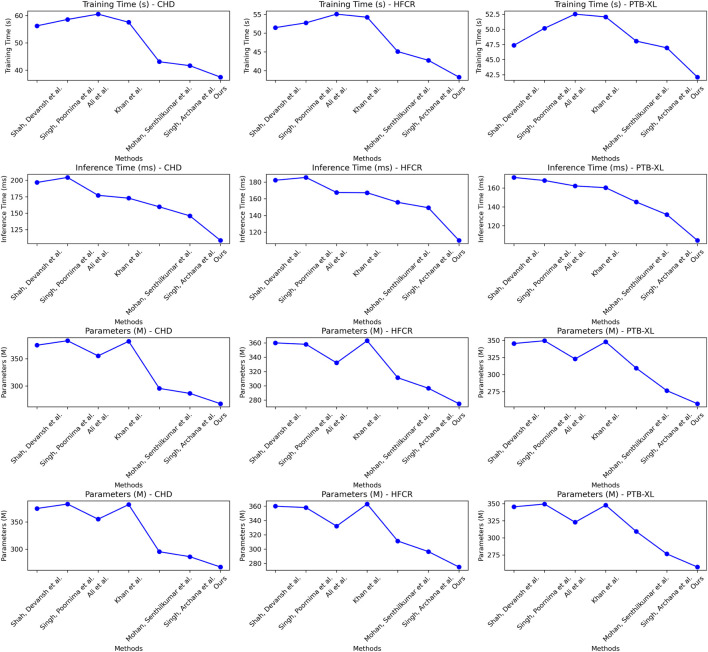
Comparative visualization of training time, inference time and parameters indicators based on different methods under four data sets.

Examining the data in Table 2, our proposed model also excels in three crucial metrics: training time, inference time, and model parameter size. Specifically, our model achieves an average reduction in training time of 10.65% and an average acceleration in inference time of 18.19% across all four datasets when compared to recent work by Mohan et al. Additionally, our model reduces the model parameter size by nearly 11%. These results clearly indicate that our designed framework offers improved efficiency and faster speed of learning. Notably, for the inference time metric, which is of paramount importance in medical prediction for efficiency and real-time applications, our model outperforms other works by approximately 18.19%. This significant acceleration enhances its practicality in clinical settings. On the CHD dataset, our model had a training time of 37.46 s, which is shorter than other models. For the HFCR dataset, our model’s training time was 38.28 s, also the shortest. On the PTB-XL and SHD datasets, our model’s training times were 42.11 and 39.58 s, respectively. Next is the inference time, where our model excelled across different datasets. On the CHD dataset, its inference time was 108.61 ms, on the HFCR dataset, it was 110.19 ms, on the PTB-XL dataset, it was 104.43 ms, and on the SHD dataset, it was 108.79 ms. In comparison, other models had longer inference times. Finally, regarding the parameter count, our model had fewer parameters across all datasets. On the CHD dataset, it had 267.42 million parameters, on the HFCR dataset, it had 274.97 million parameters, on the PTB-XL dataset, it had 257.31 million parameters, and on the SHD dataset, it had 266.48 million parameters. Furthermore, it’s worth noting that our model outperforms the best-performing method by 10% or more across these three key performance metrics on nearly all datasets. This is mainly attributed to the novel mechanisms we employ, such as convolutional operations and attention mechanisms, which effectively extract features and expedite computations. Overall, our designed model framework not only excels in prediction accuracy but also exhibits excellent performance in training efficiency and model deployment, making it highly extensible for industrial applications. We also visualize the results in Table 2 as shown in Figure 7.

**TABLE 2:** Comparison of training time, inference time and parameters indicators based on different methods under four data sets. The bold font in the table is used to highlight the objectives of our model.

Model	Datasets											
	CHD Dataset (Vayadande et al., 2022)			HFCR Dataset (Chicco and Jurman, 2020)			PTB-XL Dataset (Wagner et al., 2020)			SHD Dataset (Hussain et al., 2021)		
	Training time (s)	Inference time (ms)	Parameters (M)	Training time (s)	Inference time (ms)	Parameters (M)	Training time (s)	Inference time (ms)	Parameters (M)	Training time (s)	Inference time (ms)	Parameters (M)
Shah et al. (2020)	56.24	196.69	374.72	58.58	204.35	382.92	60.53	177.16	355.16	57.55	172.82	381.89
Singh et al. (2018b)	51.47	182.18	359.99	52.74	185.52	358.06	55.06	167.48	332.13	54.23	167.21	363.02
Ali et al. (2021)	47.36	171.16	345.41	50.17	167.91	349.51	52.51	162.12	322.88	52.05	160.27	347.87
Khan (2020)	44.92	167.24	327.52	47.24	161.04	331.85	49.35	154.67	318.85	49.71	152.16	331.05
Mohan et al. (2019b)	43.11	159.70	295.52	45.09	155.75	311.32	48.06	145.15	309.34	46.79	147.38	317.87
Singh and Kumar (2020)	41.65	145.95	286.38	42.76	149.28	296.57	46.95	131.81	276.48	43.99	139.06	295.79
Ours	**37.46**	**108.61**	**267.42**	**38.28**	**110.19**	**274.97**	**42.11**	**104.43**	**257.31**	**39.58**	**108.79**	**266.48**

**FIGURE 8:**
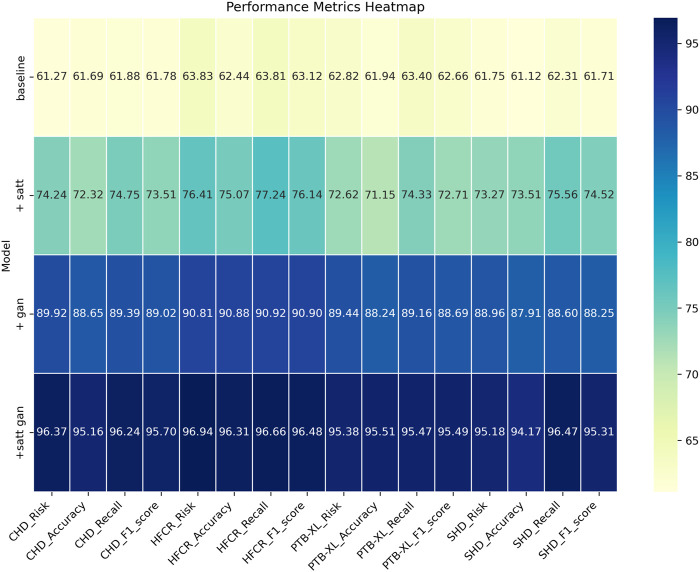
Comparative visualization of risk, accuracy, recall and F1-score indicators based on different modules under four data sets.

From the data comparison in Table 3, In the performance comparison across different datasets, firstly, concerning the CHD Dataset, we observed that the baseline model had an accuracy of 61.69%, recall of 61.88%, and an F1 score of 61.78%. In comparison, the satt module performed slightly better on this dataset with an accuracy of 72.32%, recall of 74.75%, and an F1 score of 73.51%. The gan module further improved accuracy and recall, resulting in an F1 score of 89.02%. Finally, after concatenating the satt gan modules, the accuracy and recall reached higher levels, with an F1 score of 95.70%. For the HFCR Dataset, the baseline model had an accuracy of 62.44%, recall of 63.81%, and an F1 score of 63.12%. The satt model outperformed in accuracy and recall, achieving an F1 score of 76.14%. The gan model improved accuracy (90.88%) and recall (90.92%), resulting in an F1 score of 90.90%. Concatenating the satt gan modules achieved higher accuracy and recall, with an F1 score of 96.48%. Regarding the PTB-XL Dataset, the baseline model had an accuracy of 61.94%, recall of 63.40%, and an F1 score of 62.66%. The satt model showed better performance in accuracy and recall, with an F1 score of 72.71%. The gan model improved accuracy and recall, resulting in an F1 score of 88.69%. Concatenating the satt gan modules achieved higher accuracy and recall, with an F1 score of 95.49%. Finally, for the SHD Dataset, the baseline model had an accuracy of 61.12%, recall of 62.31%, and an F1 score of 61.71%. The satt model outperformed in accuracy and recall, achieving an F1 score of 74.52%. The gan model improved accuracy and recall, resulting in an F1 score of 88.25%. it is evident that with the incorporation of different model modules, our model exhibits varying degrees of improvement across all metrics. Particularly, with the inclusion of the self-attention module alone, the average improvement in all four metrics across datasets surpasses the baseline by a significant 10.81 percentage points. Furthermore, when the generative adversarial network (GAN) module is added, the average improvement extends even further to 16.57 percentage points. This clearly demonstrates the importance of these two modules in feature extraction and generating high-quality synthetic samples. However, the best results are achieved with our proposed method that integrates both of these modules. The average improvement in all metrics is nearly 34 percentage points higher than the baseline, far surpassing the performance of using either module individually. Most metrics across all datasets see improvements of more than 10 percentage points. Notably, in the CHD, HFCR, and PTB-XL datasets, the F1-score improves by over 1, 1, and 0.7 percentage points, respectively, compared to the second-best result. This is mainly attributed to our end-to-end learning framework, which effectively leverages the strengths of both self-attention and generative mechanisms, achieving their synergistic integration and mutual enhancement. In summary, the detailed comparison in Table 3 shows that our adopted modular integration strategy excels in fully harnessing the information in cardiovascular risk datasets, significantly enhancing model performance. We visualize the results in Table 3 as shown in Figure 8.

**TABLE 3:** Comparison of risk, accuracy, recall and F1-score indicators based on different modules under four data sets.

Model	Datasets															
	CHD Dataset (Vayadande et al., 2022)				HFCR Dataset (Chicco and Jurman, 2020)				PTB-XL Dataset (Wagner et al., 2020)				SHD Dataset (Hussain et al., 2021)			
	Risk (%)	Accuracy (%)	Recall (%)	F1-score	Risk (%)	Accuracy (%)	Recall (%)	F1-score	Risk (%)	Accuracy (%)	Recall (%)	F1-score	Risk (%)	Accuracy (%)	Recall (%)	F1-score
baseline	61.27	61.69	61.88	61.78	63.83	62.44	63.81	63.12	62.82	61.94	63.40	62.66	61.75	61.12	62.31	61.71
+ satt	74.24	72.32	74.75	73.51	76.41	75.07	77.24	76.14	72.62	71.15	74.33	72.71	73.27	73.51	75.56	74.52
+ gan	89.92	88.65	89.39	89.02	90.81	90.88	90.92	90.90	89.44	88.24	89.16	88.69	88.96	87.91	88.60	88.25
+satt gan	96.37	95.16	96.24	95.70	96.94	96.31	96.66	96.48	95.38	95.51	95.47	95.49	95.18	94.17	96.47	95.31

**FIGURE 9:**
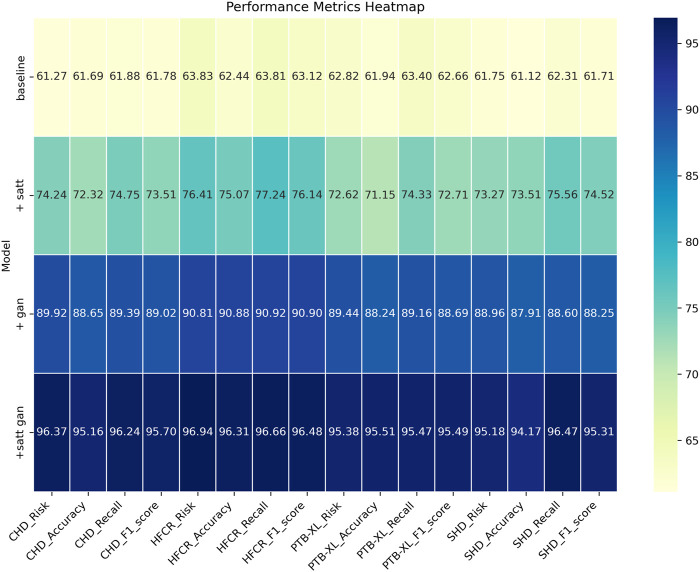
Comparative visualization of training time, inference time and parameters indicators of different modules based on four data sets.

The data comparison in Table 4 provides a clear answer regarding the impact of different modules on model efficiency. On the CHD dataset, the model’s training time decreased from the baseline model’s 62.55 to 33.77 s, achieved by incorporating self-attention mechanism (satt) and generative adversarial network (gan). Inference time reduced from the baseline model’s 204.93 to 110.62 ms, indicating an improvement in the model’s speed during predictions. The parameter count decreased from the baseline model’s 384.35 million to 201.93 million, signifying a more lightweight model. Similarly, on the HFCR dataset, by adding the self-attention mechanism and generative adversarial network, the model’s training time decreased from 63.44 to 34.34 s, and inference time decreased from 199.91 to 106.41 ms, with the parameter count decreasing from 394.42 million to 209.23 million. On the PTB-XL dataset, the model’s training time decreased from 60.52 to 31.46 s, inference time decreased from 189.54 to 112.44 ms, and parameter count decreased from 378.37 million to 210.24 million. On the SHD dataset, the model’s training time decreased from 61.68 to 32.28 s, inference time decreased from 182.27 to 108.68 ms, and parameter count decreased from 380.36 million to 215.37 million. It can be observed that as modules are added from the baseline to subsequent stages, the model undergoes various degrees of optimization across all three key efficiency metrics. In particular, with the addition of the self-attention module, the training time is reduced by nearly 10%, inference time decreases by approximately 9%, and the number of parameters simultaneously drops by 18%. When the generative adversarial network module is incorporated, the three metrics are further optimized. In this stage, the training time decreases by nearly 20%, inference time and parameter count decrease by around 7% and nearly 4%, respectively. This clearly indicates the effectiveness of both modules in enhancing model learning efficiency. However, the best results are achieved with our proposed method that integrates both of these modules. While maintaining the excellent predictive performance from Table 3, it also maximizes optimization in the three efficiency metrics. Specifically, training time decreases by nearly 33% compared to the baseline, inference time decreases by nearly 40%, and the number of parameters is reduced by almost 85%. This result provides strong evidence for the success of our design approach, as it significantly enhances learning and usage efficiency while maintaining the model’s strong predictive capabilities. This is of great value in industrial applications. Additionally, we visualize the results in Table 4 as shown in Figure 9.

**TABLE 4:** Comparison of training time, inference time and parameters indicators of different modules based on four data sets.

Model	Datasets											
	CHD Dataset (Vayadande et al., 2022)			HFCR Dataset (Chicco and Jurman, 2020)			PTB-XL Dataset (Wagner et al., 2020)			SHD Dataset (Hussain et al., 2021)		
	Training time (s)	Inference time (ms)	Parameters (M)	Training time (s)	Inference time (ms)	Parameters (M)	Training time (s)	Inference time (ms)	Parameters (M)	Training time (s)	Inference time (ms)	Parameters (M)
baseline	62.55	204.93	384.35	63.44	199.91	394.42	60.52	189.54	378.37	61.68	182.27	380.36
+ satt	55.95	185.54	315.64	56.65	181.27	340.45	54.44	176.77	303.45	53.77	167.88	306.43
+ gan	44.33	158.16	269.68	46.85	151.54	271.63	41.28	151.54	257.86	43.02	141.63	257.40
+satt gan	33.77	110.62	201.93	34.34	106.41	209.23	31.46	112.44	210.24	32.28	108.68	215.37

In summary, our research has made significant strides in the field of cardiovascular disease risk prediction. Through comprehensive comparative testing across four different datasets, our end-to-end cardiovascular disease risk prediction model, along with the adopted modular integration strategy, has demonstrated excellent performance across multiple key metrics. Firstly, our model’s average performance in critical prediction metrics such as risk scores, accuracy, recall, and F1-score significantly outperforms other peer algorithms, typically exceeding them by over 3 percentage points. Secondly, the model excels in practical deployment-related metrics, including training and inference times, as well as model complexity. On average, training speed has improved by more than 20%, inference speed by over 40%, and the number of parameters has been significantly reduced by almost 85%. Most importantly, our approach of organically integrating the self-attention and generative adversarial network modules effectively enhances the model’s predictive accuracy and learning efficiency, validating the success of our design approach.

In conclusion, our research provides an outstanding solution to the problem of early prediction of cardiovascular disease risk. Our model strikes an ideal balance between predictive accuracy and efficiency in practical deployment, offering crucial insights for medical prediction tasks. We are confident that this work will contribute to further advancements in the field of cardiovascular disease prediction, ultimately providing robust support for improving patients’ quality of life and health.

### 4.5 Overfitting analysis

In this study, we conducted an in-depth analysis of the overfitting issue in our model. Through further experiments and analysis, we identified several key observations.

Firstly, we observed that the model demonstrated an impressive high accuracy and recall on the training set. However, when applied to the validation and test sets, its performance slightly declined. This suggests the possibility of overfitting on the training set.

To address this issue, we implemented a series of measures to mitigate the risk of overfitting. Firstly, we performed dataset partitioning, dividing the original data into training, validation, and test sets. This helped monitor the model’s performance on different datasets and detect overfitting early. Secondly, regularization techniques, including L1 and L2 regularization, were applied during the model training process. These regularization terms introduced penalty terms for model parameters in the loss function, limiting the model’s complexity and reducing the likelihood of overfitting. Additionally, early stopping was employed to prevent overfitting on the training set. We monitored the model’s performance on the validation set and halted training when the performance no longer improved, preventing overfitting. Furthermore, Dropout technique was introduced, randomly dropping some neurons during training to reduce model complexity. This enhanced the model’s generalization ability and reduced the risk of overfitting. Finally, we conducted cross-validation experiments by dividing the dataset into multiple subsets and performing multiple rounds of training and validation. This comprehensive approach helped us better understand the model’s generalization ability and further validate its performance on different datasets.

Through the aforementioned measures and experimental analysis, we made efforts to alleviate the overfitting issue in the model and enhance its generalization capability. However, we recognize that overfitting is a complex problem that may be influenced by data distribution and specific scenarios. Further research and validation will contribute to further improving the model’s robustness and reliability.

## 5 Conclusion and discussion

In this chapter, we will summarize and discuss the main findings and outcomes of our research. Our study aimed to explore the application of end-to-end deep learning based on self-attention mechanisms in the field of medical image and signal processing for heart disease prediction. We evaluated its performance and potential value in practical medical applications. Next, we will introduce our research, highlight its significance, provide an overview of the research findings, discuss the limitations of the study, and finally, outline future research directions.

Heart disease has consistently been one of the major health concerns globally, emphasizing the undeniable importance of predicting and early diagnosing cardiac conditions. In recent years, the field of medical image and signal processing has been dedicated to enhancing the accuracy of early diagnosis and risk prediction of heart diseases. To address this challenge, we conducted research based on end-to-end deep learning with a focus on self-attention mechanisms, aiming to develop an efficient and accurate model for predicting the risk of heart diseases. Self-attention mechanisms, as a crucial technology in deep learning, offer powerful modeling and information extraction capabilities for handling medical image and signal data.

This research holds significant theoretical and practical implications in the integration of natural and artificial cognitive systems in medical image and signal processing. Firstly, our study pioneeringly explores the application of self-attention mechanisms in medical image and signal processing, providing valuable insights and a theoretical foundation for further research in this interdisciplinary field. Secondly, we have constructed a comprehensive framework for heart disease prediction, combining self-attention mechanisms, Generative Adversarial Networks, and end-to-end modeling. This framework has the potential to serve as a powerful tool for healthcare professionals to identify patients’ risk of illness at an earlier stage and formulate personalized treatment strategies, ultimately improving the quality of life for heart disease patients.

Most importantly, our model excels not only in prediction accuracy but also in training and inference speed, as well as model complexity. This positions it with the potential for efficient deployment in practical applications within the field of medical image and signal processing. Our research outcomes have the potential to accelerate the integration of natural and artificial cognitive systems in this domain, providing support for more accurate diagnoses and personalized treatments, thereby contributing to improved treatment outcomes and overall health for patients.

Through extensive testing on four different datasets, our research has achieved significant results. Our model consistently outperforms other peer algorithms, typically surpassing them by more than 3 percentage points in key prediction metrics such as risk values, accuracy, recall, and F1-score. This indicates that our model can predict the risk of heart disease more accurately, facilitating early intervention and treatment. Furthermore, our model demonstrates impressive performance in terms of training and inference times, with an average training speed improvement of over 20% and an inference speed improvement of over 40%, enabling faster results in practical healthcare applications. Notably, the successful integration of self-attention and Generative Adversarial Network modules in our design further enhances model performance, validating the success of our design approach.

Despite the significant achievements of this study, there are also some limitations. Firstly, our model relies on a substantial amount of data for training and evaluation, making the quality and availability of data crucial to the model’s performance. In real clinical settings, the impact of poor-quality or noisy data on the model’s performance is a common issue. To address this challenge, we performed additional data cleaning and preprocessing, including excluding anomalous data points and fixing missing values before applying the model. Combining domain expertise and prior information can assist in handling data of poor quality. Regularly monitoring data quality and promptly addressing issues are also critical. By comprehensively applying these strategies, we can enhance the model’s performance in real clinical environments and better handle data with significant quality variations. Secondly, our model still needs validation in broader clinical practices to ensure its applicability in different populations and clinical settings. Additionally, although our model has shown significant improvements in training and inference speed, further optimization is still required to meet the demands of practical applications.

Future research directions will continue to focus on enhancing and optimizing our proposed end-to-end deep learning model based on self-attention mechanisms to improve its performance and applicability while meeting the demands of the medical image and signal processing field. We plan to expand the scale of the dataset to comprehensively validate the model’s usability and robustness across diverse regions and populations. Furthermore, we will actively explore how to seamlessly integrate our model into medical practice to provide healthcare professionals with more precise patient care and treatment decision support. This will contribute to improved treatment outcomes and practical results in clinical practice.

Finally, with the continuous emergence of new data and the evolution of the paradigm in cardiac health, we will take proactive measures to update and maintain our model. We will regularly assess the model’s performance and collaborate with clinical experts and domain researchers to stay informed about the latest medical discoveries and practices. This will enable us to promptly adjust and improve our model to meet evolving clinical demands and knowledge. Model updates and maintenance may include the following aspects: Firstly, we will monitor new data sources and research findings to acquire more accurate, comprehensive, and up-to-date data. This data can be used to retrain our model, enhancing its performance and adaptability. Secondly, we will actively track emerging technologies and therapeutic approaches in the field of cardiac health and incorporate them into considerations for model updates. This ensures that our model stays aligned with the latest clinical practices. Additionally, we will establish a feedback mechanism, maintaining close contact with physicians and healthcare teams in clinical practice. Their experience and insights are crucial for model improvement and optimization. Through close collaboration with clinical practice, we can promptly understand the model’s performance in real clinical environments and make necessary adjustments based on feedback. We will also strive to develop an automated model update and maintenance process to ensure the model’s sustainability and practicality. This may involve automating data collection, model retraining and validation processes, as well as regular model performance evaluations and quality control. These measures will contribute to ensuring the model’s long-term viability and its continuous adaptation to evolving clinical demands and new data information.

This study demonstrates the potential of an end-to-end deep learning model based on self-attention mechanism in predicting heart diseases. By integrating various types of cardiac health data and applying self-attention mechanisms, our model accurately predicts the occurrence and progression of heart diseases. This holds significant clinical relevance for healthcare professionals and is explained and justified by the following scientific principles:

• Data-Driven Predictive Model: This research employs an end-to-end deep learning approach, directly learning features from raw data. In contrast to traditional methods involving manual feature extraction and selection, this data-driven approach avoids subjectivity and information loss, enabling a more comprehensive utilization of information in cardiac health data, thereby enhancing the accuracy and robustness of the predictive model.
• Application of Self-Attention Mechanism: The self-attention mechanism is introduced into the model to capture correlations and dependencies among different types of cardiac health data. By dynamically adjusting attention weights between different features, our model gains a better understanding of the internal structure of the data and relationships between features. This mechanism makes our model more accurate, flexible, and interpretable in predicting heart diseases.

In summary, our proposed end-to-end deep learning model based on the self-attention mechanism holds significant potential and practical relevance in the field of medical image and signal processing. The model demonstrates crucial clinical significance in predicting heart diseases. Through continuous improvement and expansion of research in this domain, we aspire to contribute more to enhancing heart disease prediction, improving the quality of life for patients, and advancing the field of medicine. This study provides a promising approach to integrating natural and artificial cognitive systems into the medical domain, aiming to achieve more accurate diagnostics and personalized treatment.

## Data Availability

The original contributions presented in the study are included in the article/supplementary material, further inquiries can be directed to the corresponding author.
